# Common Accidents of Every-Day Life

**Published:** 1887-12-31

**Authors:** Robert A. P. Forrester


### The Common Accidents of Every-day Life.

By Robert A. P. Forrester, B.A.Dub., M.B. and C.M.Edin.

**Burns and Scalds**—(*continued*).

*Treatment.*—This is of two kinds—local and constitutional First as to the local treatment. I take it for granted that in the case of the clothes catching fire the blaze has been put out. This is best done by throwing the patient on the floor, wrapping him up in the folds of a hearthrug, table cover, or curtains—do not let him run about for obvious reasons. Cut away the clothing from the injured part, taking care not to tear the skin, especially if blisters have formed. Should there be much prostration, give some stimulant.

In burns of the first degree, cold applications are most agreeable. Goulard's water is a favourite and very efficient remedy in hospitals. Soak some rags in this, and keep them constantly wet with the lotion. The great thing is to exclude the air, and where there is a good deal of depression from the pain and fright, a good thing to do is to dredge the injured parts with flour and cover over carefully with cotton-wool. The new cuticle will have formed in a day or two, and nothing further is necessary.

In burns of the second degree, where we have vesicles, let out the fluid very carefully, and press the cuticle gently down upon the skin.

Burns of the third degree are most serious, for here some of the true skin has been destroyed, and sloughing must follow. A stimulating application is useful in order to hasten the separation of the slough. A very good one is carron oil—(equal parts of linseed oil and lime water). This oil is so called from Carron, in Stirlingshire, where it was much used by workmen employed in the foundries. *Carronades*, a small kind of cannon, used to be manufactured there. Carbolic oil is also an excellent remedy, but if the burn be extensive absorption may occur from its use. Turpentine liniment has given good results, but where the parts are raw and tender it would be a painful application. To apply any of the above, soak some lint in the oil, and cover over carefully with cotton-wool, and bandage the whole lightly.

The dressings ought not to be changed too frequently; allow the first dressing to remain twenty-four hours, and then it should be repeated only as often as cleanliness demands. The sloughing skin ought to be cut away gently with a pair of scissors, and when the surface is left raw and suppurating the ordinary treatment for ulcers may be adopted.

In burns of the fourth degree the above treatment may also be used, but during the healing process there is danger of contraction and consequent deformity occurring. Various kinds of extending apparatus have been adopted for the prevention of such a result. It is not uncommon to see the head drawn down upon the shoulder, the lips averted, rigid limbs, and other ghastly mutilations after a scald or a burn has healed.

Burns of the fifth and sixth degree are generally fatal. If the burn is localised in a limb the usual treatment adopted is amputation.

The constitutional treatment mainly consists in husbanding the strength and meeting symptons as they arise, and must be under the direction of a medical man.

**Bones, Fractures, Dislocations**—(*continued*).

It would fill a small volume to describe all the kinds of splints in use, *e.g.*, Macintyre's, Paget's, Liston's, Dupuytren's, the stirrup splint, pistol splint, etc., but reference can be made to them afterwards when speaking of particular fractures.

A fracture generally unites in from five to six weeks, but is never completely consolidated until about seven weeks at least after the accident. It is still soft, and would readily yield if any pressure were put on the limb. At this time, after the removal of the splints, it is safer to apply some retentive apparatus—such as a starch bandage. When the thigh is broken no attempt should be made to use it for some time after the splint has been removed. In the forearm, the splints may be removed after three or four weeks, and passive movement begun to prevent thickening and adhesions of tendons to their sheaths.

*General Treatment of Dislocations.*—The first indication is to reduce the bone, that is, to put it in its place again, and the sooner this is done the better. For this purpose we employ manipulation or some mechanical contrivance. To manipulate a bone into its old position scientifically, and with little pain to the patient, requires a knowledge of anatomy and surgery. The "bone-setter" may accomplish this by good luck or the employment of brute force, but his end is often attained at the expense of great suffering, and perhaps of the limb itself. The surgeon with the requisite knowledge lifts the limb gently in his hands, and guides it promptly, perhaps, into its socket. Every surgeon, however, does not possess this facility—it is only gained by study, experience, and a familiarity with anatomy and the functions of the various muscles.

Without the possession of these qualifications the operator had better leave the treatment of dislocations alone, for he will constantly be in danger of mistaking a dislocation for a fracture, and *vice versa.* The most experienced surgeons are liable to error; how much more is the ignorant one?

If manipulation fail, we then try extension and counter-extension, with the aid, of course, of an assistant. The surgeon seizes the limb, firmly extends it, and the assistant pulls in the opposite direction. We may combine manipulation with this, and in addition employ pressure upon the head of the bone itself. Pulleys have also been employed when this has failed.

*Repair of Broken Bones.*—It was by the examination of recently-united fractures in persons who had died suddenly from other causes, of old fractures, and also by experiments on animals, etc., that surgeons came to conclusions regarding the process of repair. Old Arabian physicians wrote of the ossifying juices that were poured out from the broken bone—the *callus*, as it was named. Others regarded it as something "poured out like lead from a plumber's ladle."

The following is what is believed to take place: The repairing material or, as surgeons say, *plastic lymph*, is poured out from the vessels of the Haversian canals, the marrow, the periosteum, and the lacerated tissues around. After some time this lymph becomes organized, and begins to receive from the same sources bony particles, and finally the union is effected. When a fracture is *well set, i.e.*, when the broken ends are accurately adjusted, there ought only to be a line marking where the fracture has occurred, with little or no thickening around. In badly-adjusted fractures, however, where there is over-lapping, etc., there is thickening, and in many cases actual deformity. Hence the popular notion that the bone is stronger after a fracture than before.

The "setting" of a broken bone is a business requiring a high degree of technical skill for its proper accomplishment, and should on no account be attempted by anyone except a duly qualified and experienced practitioner. Amateurs of all kinds, including bone-setters, are very dangerous persons.

(*To be continued.*)

